# How to write a successful graduate school application

**DOI:** 10.1038/s44319-023-00001-9

**Published:** 2023-12-14

**Authors:** Shina Caroline Lynn Kamerlin

**Affiliations:** Georgia Institute of Technology, Atlanta, GA USA

## Abstract

Practical advice for prospective graduate students on how to apply for a PhD position in the USA and Europe.

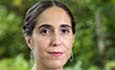

It is that time of the year again: graduate school applications to STEM programs in the United States are in full swing, and prospective candidates are busy applying to schools across the country. I have, in the past few weeks, received several emails from students asking me what they can do to write a more competitive application and I am distilling here the advice I have been sharing with them. While this is primarily focused on the US graduate school process, the principles are the same: I have personally evaluated students both in Europe and in the United States, and although the administrative processes are different, the core components of a successful application are not. Although my focus here is on admittance to a PhD program, my advice may also serve applicants for a master’s program, postdoctoral positions, or faculty positions.

There are some significant differences in the application process between European and US universities. In Europe, universities typically require a research-intensive master’s degree before considering students for a PhD program, whereas in the United States, a master’s degree is not normally a prerequisite. Further, in Europe, with some exceptions, most applicants will directly contact a potential advisor who has announced a PhD position in their group, and this may happen at any time of the year. In the United States, you usually apply to a PhD program at the departmental level, and then select an advisor after admittance and after talking to the faculty and participating in short rotations in their research labs. This follows a specific calendar, with many programs having December 1st deadlines for priority consideration. Non-US applicants have to take an English Language Proficiency test as part of the application, and US graduate programs typically have an application fee, although this can be waived under certain circumstances.

Beyond these structural and administrative differences, however, the core components of a graduate school application in the United States and in Europe are very similar: a personal statement/cover letter, transcripts, letters of recommendation, and sometimes samples of work. In Europe, you might also submit an electronic copy of your Master’s thesis as part of your application, and in the United States, you could, for instance, upload copies of publications you have published as an undergraduate or other pertinent material. These will be scrutinized carefully by the admission committee, whether this scrutiny is done by a potential graduate advisor in Europe with colleagues assisting, or by a departmental admission committee in the United States.

It is obvious that a strong transcript is necessary for a competitive PhD program, and that additional factors such as publications will further help in the application process. However, two very important components are the letters of recommendation, and the personal statement/cover letter. In the case of letters of recommendation, think carefully about whom you ask to write letters for you. How well do you know them professionally? Do they know enough about you to do you justice? Is this someone who will write a strong letter highlighting your best features professionally, and treat fairly areas of growth? It is also very useful, if possible, to have letters of recommendation from different areas: either different subjects, and/or different forms of professional involvement, such as research versus classes versus TA experience, as relevant. This highlights your versatility, and how you are performing within multiple areas, in addition to your academic and research merits.

The next most important component is your personal statement. Your transcript and related achievements are a record of what you have achieved academically. Your cover letter is your opportunity to show the selection committee who you are, what you are passionate about, and why you are excited about that program. I cannot emphasize enough the importance of investing time into this document and tailoring it to the institution you are applying to. The graduate admission process is competitive, and you will need to apply to multiple programs to be successful. However, it is important you target your letter individually to the institution you are applying to. What you want to convey is a sense of genuine excitement about the program and why you want to join it, what you would bring to the program in terms of your background, experiences, and training, and also why you think this program is a good fit for you and what you hope to gain from it.

Some (but not all) US programs will have an interview stage, whereas interviews are the norm for a European graduate admission process. In the event you are shortlisted for an interview, these are points you also want to bring across. Do your homework, learn about the department, what sort of research is done there, and it helps also to identify potential advisors you would want to work with if admitted. In the case of European applications where you apply directly to an advisor, study the advisor’s publication track record, learn what they are excited about and what work they are doing, and make a case for how you would fit into that program. It is also okay to identify potential faculty you would like to work with and reach out to them ahead of your application and ask to discuss further. This is in fact quite important in terms of creating the foundations for a professional relationship and can provide leverage in admission circumstances. Essentially, and most importantly, you want to convey genuine interest and commitment to the program.

Finally, once you are admitted, take your time to make sure this program is the best match for you. Take opportunities to visit the campus and meet the faculty—or ask for such an opportunity if not directly offered—talk to current and if possible former graduate students at the department and see if this is a place you think is a good fit. Also, talk to the graduate students in the lab you are interested in. It is important to know how they feel working under their advisor and if the work environment is going to correlate with you. One of the things I like most about the US PhD training is the opportunity to do lab rotations and working in different labs before mutually settling on the advisor and lab you will undertake your thesis work in. This gives you maximum flexibility to settle on an institution and advisor that will be the best fit for you and for your training goals.

Graduate school admissions are a complex and often competitive process. My advice above is not a surefire path to admission. But it will hopefully help you to construct the strongest-possible case for why *you* are the candidate that the committee should be excited about and, if relevant, preparing for your interviews. If you are reading this because you are in the process of applying to a graduate school, good luck!

